# Federal Look-Alike Plan Termination Policy and Dual-Eligible Enrollment in Integrated Care Programs

**DOI:** 10.1001/jamahealthforum.2025.6294

**Published:** 2026-01-16

**Authors:** Yanlei Ma, Eric T. Roberts, Jessica Phelan, Kenton J. Johnston, E. John Orav, Ellen R. Meara, Jose F. Figueroa

**Affiliations:** 1 Department of Health Policy and Management, Harvard T.H. Chan School of Public Health, Boston, Massachusetts; 2 Perelman School of Medicine, University of Pennsylvania, Philadelphia; 3 Department of Medicine, Washington University in St Louis, Missouri; 4 Department of Medicine, Brigham and Women’s Hospital, Boston, Massachusetts

## Abstract

**Importance:**

In 2023, the Centers for Medicare & Medicaid Services terminated dual-eligible special needs plan look-alikes—Medicare Advantage plans with beneficiary panels composed of more than 80% dual-eligible individuals but lacking Medicaid integration. Understanding whether this policy promoted dual-eligible enrollment in integrated care plans, particularly those attaining high-level integration, is critical.

**Objective:**

To describe dual-eligible enrollment transitions after the look-alike plan termination and evaluate whether the policy was associated with increased enrollment in highly integrated plans.

**Design, Setting, and Participants:**

This repeated cross-sectional study analyzed US Medicare administrative data from January 2017 to January 2023. Samples were limited to full-benefit dual-eligible beneficiaries.

**Main Outcomes and Measures:**

First, a beneficiary-level analysis was conducted on 2023 enrollment patterns among full-benefit dual-eligible individuals whose 2022 plans were terminated, including factors associated with enrollment in highly integrated plans in 2023. Next, a county-year–level difference-in-differences design was used to compare changes in full-benefit dual-eligible enrollment before (2017-2022) and after (2023) the termination policy between counties with vs without terminated look-alike plans. A difference-in-differences design was used to evaluate whether the look-alike termination policy was associated with the proportion of full-benefit dual-eligible individuals enrolled in highly integrated care plans.

**Results:**

Between 2017 and 2022, 482 of 2576 counties had full-benefit dual-eligible individuals enrolled in look-alike plans for at least 1 year. Of the 170 399 full-benefit dual-eligible individuals enrolled in look-alike plans in 2022 (58.9% female; 20.6% Asian, 44.8% Hispanic, 11.3% non-Hispanic Black, 21.4% non-Hispanic White, and 2% other) and remained dual-eligible in 2023, only 5.4% transitioned to highly integrated plans, while 55.6% moved to nonintegrated plans. Dual-eligible individuals transitioning to highly integrated plans were more likely to be older (65-74 years: adjusted difference, 3.4 percentage points [pp]; 95% CI, 2.8-4.1 pp; 75-84 years: adjusted difference, 4.1 pp; 95% CI, 3.3-4.8 pp; ≥85 years: adjusted difference, 5.0 pp; 95% CI, 4.0-5.9 pp), female (adjusted difference: 0.6 pp; 95% CI, 0.2-0.9 pp), without disabilities (adjusted difference, −0.7 pp; 95% CI, −1.2 to −0.2 pp), and less likely to be Asian (adjusted difference, −5.0 pp; 95% CI, −5.6 to −4.4 pp) or Black (adjusted difference, −0.9 pp; 95% CI, −1.6 to −0.2 pp). The termination policy was not associated with a significant differential increase in enrollment into highly integrated plans in counties with look-alike plans compared with those without (0.6 pp; 95% CI, −0.4 to 1.6 pp). However, there was a 2.6-pp differential increase (95% CI, 0.01-5.1 pp) in enrollment into plans offering some integration, primarily driven by enrollment growth in plans with lower levels of integration. Enrollment also increased in conventional Medicare Advantage plans with fewer than 80% of dual-eligible enrollees (2.6 pp; 95% CI, 0.7-4.5 pp) after the termination policy.

**Conclusions and Relevance:**

In this study, the termination of look-alike plans was insufficient to significantly shift dual-eligible individuals toward highly integrated plans. Complementary strategies are necessary to ensure that dual-eligible individuals enroll into highly integrated care models that may improve outcomes.

## Key Points

**Question:**

Is the federal policy eliminating nonintegrated dual-eligible special needs plan look-alike plans associated with increased enrollment of Medicare-Medicaid dual-eligible beneficiaries into integrated care plans, particularly those with high-level Medicare-Medicaid integration?

**Findings:**

This repeated cross-sectional study using Medicare data on full-benefit dual-eligible individuals in 2576 US counties between 2017 and 2023 found that the termination of look-alike plans was not associated with any increase in dual-eligible enrollment into plans with high-level integration. The majority of dual-eligible individuals in terminated look-alike plans transitioned into nonintegrated plans.

**Meaning:**

Results of this study suggest that policymakers should consider additional strategies beyond the look-alike termination policy to expand dual-eligible enrollment in highly integrated plans.

## Teaser

This repeated cross-sectional study evaluates whether the federal policy eliminating nonintegrated dual-eligible special needs plan look-alike plans increased enrollment of Medicare-Medicaid dual-eligible beneficiaries into integrated care plans.

## Introduction

Approximately 12 million individuals are dually eligible for and enrolled in both Medicare and Medicaid (dual-eligible individuals).^1^ These individuals have complex medical, long-term care, and health-related social needs, and they incur nearly one-third of the health care spending across both programs.^2^ Federal and state policymakers have been prioritizing efforts to expand integrated care models for dual-eligible beneficiaries to better coordinate care across Medicare and Medicaid.^3^ Currently, the largest managed care model for dual-eligible individuals is the dual-eligible special needs plan (D-SNP). D-SNPs are Medicare Advantage (MA) plans required to contract with state Medicaid programs to coordinate Medicare and Medicaid benefits, although the degree of care coordination and financial integration varies considerably among these plans.^3,4^ Although empirical evidence remains limited, research suggests that integrated care plans responsible for both Medicare and Medicaid spending may improve care coordination and efficiency in certain areas, such as long-term care.^5,6^

In recent years, however, a threat to federal and state integrated care policy has emerged in the form of D-SNP look-alike plans, which are conventional nonintegrated MA plans that disproportionately enroll dual-eligible individuals.^7,8,9,10^ Although look-alike plans, as with all MA plans, receive higher risk-adjusted capitation payments for dual-eligible enrollees, risk adjustment is imperfect. Plans may anticipate that actual plan costs for these patients are lower than risk-adjusted payments, particularly because many services used by dual-eligible individuals—such as long-term care—are financed by Medicaid. This creates opportunities for plans to benefit financially while shifting costs to Medicaid, raising concerns that look-alike plans weaken integration efforts because these plans are not subject to the requirements of D-SNPs to coordinate Medicare and Medicaid services. Prior research documented rapid growth in dual-eligible enrollment in look-alike plans, particularly among Hispanic beneficiaries and those in socially vulnerable communities.^7,10^

To address this issue, effective January 2023, the Centers for Medicare & Medicaid Services (CMS) terminated contracting with any conventional nonintegrated MA plans where 80% or more of plan enrollees are dual-eligible beneficiaries, including those with either full or partial Medicaid benefits.^11^ Subsequently, CMS further tightened the threshold to 70% in 2025 and 60% in 2026 and beyond.^12^ This action marks a unique instance where CMS unilaterally ended contracting with insurers based solely on a numerical enrollment threshold for a specific population of Medicare beneficiaries. Evaluating the initial look-alike termination policy is critical for determining whether it is an effective lever for curbing look-alike plans and advancing other priorities of the Medicare program. While a recent study^13^ documented subsequent insurance enrollment patterns following the termination of the look-alike plans, to our knowledge, no prior study has examined whether this policy was associated with increased dual-eligible enrollment into integrated care programs relative to a counterfactual.

Using national Medicare data from 2017 to 2023, this study had 2 objectives. First, we described enrollment in look-alike plans leading up to and following implementation of the look-alike termination policy, including transitions among dual-eligible individuals previously enrolled in affected plans and beneficiary-level factors associated with subsequently transitioning into integrated care plans. Second, we used a difference-in-differences (DID) design to evaluate whether the look-alike termination policy was associated with increased dual-eligible enrollment into integrated care plans—particularly plans with a high level of financial integration—in counties where the policy led to plan terminations, using counties without terminated plans as controls for secular trends.

## Methods

### Data

This repeated cross-sectional study used 100% Medicare Beneficiary Summary Files (MBSF) between January 2017 and January 2023, which contain information on beneficiary demographic characteristics, plan enrollment, and county of residence.^14^ These data were linked with Medicare Plan Characteristics Files, which report plan service areas.^15^ We followed the Strengthening the Reporting of Observational Studies in Epidemiology (STROBE) reporting guideline. To analyze factors associated with transitions into highly integrated plans following the 2023 look-alike termination among beneficiaries enrolled in look-alike plans in 2022, we used 2022 MA encounter data^16,17,18^ to calculate Hierarchical Condition Category (HCC) risk scores.^19^ Finally, we used the Centers for Disease Control and Prevention’s Social Vulnerability Index to characterize the socioeconomic vulnerability of counties where beneficiaries resided.^20^ This study was approved by the Harvard T.H. Chan School of Public Health Institutional Review Board and the requirement for informed consent was waived because the data used were deidentified.

### Identification of Look-Alike Plans

In each study year, we identified look-alike plans as conventional nonintegrated MA plans in which dual-eligible enrollees (including beneficiaries eligible for both full and partial Medicaid) constituted more than 80% of total enrollment. Dual-eligibility status and enrollment were determined annually using January enrollment information from the MBSF. Each plan was uniquely identified by its contract number and plan benefit package number. Consistent with CMS guidelines, special needs plans (SNPs), MA plans that were active for less than 1 year and with 200 or fewer total Medicare enrollees, as well as MA plans offered in states without D-SNPs, were not subject to the termination policy and therefore were excluded from the definition.^11^ Employer plans, cost plans, and Medicare savings account plans were also excluded.

### Integrated Care Plan Categorization

We followed the Medicaid and Children’s Health Insurance Program Payment Access Commission framework for classifying plans into 3 levels of integration—high, moderate, and low—according to the degree of care coordination and financial responsibility for managing Medicare and Medicaid benefits.^4^ Plans attaining high-level integration fully integrate Medicare and Medicaid services and financing under a single entity, including long-term care services and behavioral health services. These plans include the Program of All-Inclusive Care for the Elderly, state Medicare-Medicaid plans, and fully integrated dual-eligible SNPs.^4^ The Program of All-Inclusive Care for the Elderly serves individuals aged 55 years and older who need nursing home–level care and provides comprehensive medical and social services through community-based day centers.^2^ Medicare-Medicaid plans, created under the Financial Alignment Initiative, are being phased out by 2025, with many expected to transition into D-SNPs under new CMS regulations.^4^ Plans with moderate-level integration, known as highly integrated D-SNPs, coordinate benefits across both programs, manage Medicare spending, but typically bear financial responsibility for only 1 major Medicaid benefit category (either long-term care services or behavioral health services, but not both).^4^ In contrast, plans with low-level integration, known as coordination-only D-SNPs (Co-D-SNPs), are only responsible for coordinating services between programs. Co-D-SNPs manage Medicare spending, but the plans assume no financial responsibility for Medicaid services.^4^

### Study Samples and Analysis Levels

We analyzed 2 samples, 1 at the individual beneficiary level and 1 at the county-year level, corresponding to our 2 study objectives. Both samples were limited to full-benefit dual-eligible beneficiaries, as they comprise approximately three-quarters of the national dual-eligible population and have been a primary target of policy efforts to expand enrollment in integrated care plans.

First, we conducted a beneficiary-level analysis of full-benefit dual-eligible beneficiaries enrolled in look-alike plans in 2022 that were terminated in 2023. We compared the characteristics of beneficiaries who transitioned into integrated care plans with those who moved to nonintegrated plans, conditional on them surviving and remaining eligible for full Medicaid during 2023.

Second, we conducted a county-year–level analysis capturing the proportions of full-benefit dual-eligible individuals with different forms of coverage. Counties where look-alike plans were offered in any year before 2023 were classified as intervention counties, whereas counties without any look-alike plans during the same period and thus not subject to the termination policy were classified as control counties. Counties in states without major Medicare-Medicaid integrated care programs (ie, Medicare-Medicaid plans or any form of D-SNPs) for any part of the study period were excluded from the analyses, as MA plans in these states were not affected by the termination policy.^11^

### Outcomes

For the beneficiary-level analysis, we examined whether full-benefit dual-eligible individuals transitioned into integrated care plans after their look-alike plans were terminated in 2023. For the county-year–level analysis, we examined the annual proportions of full-benefit dual-eligible individuals enrolled in each plan type in each county.

The primary outcome was enrollment in plans with high-level integration. Secondary outcomes included enrollment in the following: (1) plans with moderate- or low-level integration; (2) plans with any level of Medicare-Medicaid integration (high, moderate, or low); (3) chronic condition SNPs (C-SNPs; plans designed for individuals with specific chronic conditions, which are increasingly enrolling full-benefit dual-eligible individuals but not required to coordinate services between Medicare and Medicaid)^21,22^; (4) institutional SNPs (I-SNPs; for individuals who reside or are expected to reside in long-term care facilities), (5) conventional nonintegrated MA plans with dual-eligible enrollment below 80%; and (6) traditional fee-for-service Medicare.

### Statistical Analysis

We conducted 2 sets of analyses. First, we conducted descriptive analyses at the beneficiary level to examine enrollment in look-alike plans between 2017 and 2023, as well as enrollment patterns after the look-alike termination policy took effect. Specifically, we calculated the proportion of full-benefit dual-eligible individuals in look-alike plans in 2022 who transitioned into each plan type in 2023. We estimated 2 beneficiary-level logistic regression models to examine factors associated with enrollment into plans with high vs moderate- or low-level integration (separate models for each outcome). Explanatory variables included age, sex, race and ethnicity, original reason for Medicare entitlement, HCC risk score, and county fixed effects. Race and ethnicity were defined using the Research Triangle Institute race code variable,^23^ and were analyzed given known differences in plan enrollment patterns across racial and ethnic groups.^7^

Second, we used a DID design to compare changes in full-benefit dual-eligible enrollment at the county-year level before (2017-2022) and after implementation of the look-alike termination policy (2023) between counties with and without look-alike plans. For each of the 6 outcomes, we estimated a linear regression model that included an indicator for post–policy year (2023), an indicator for intervention counties in post–policy year, county fixed effects, and time-varying county-level full-benefit dual-eligible characteristics (including age distribution [aged 65-74, 75-84, and ≥85 years], sex [male], race and ethnicity [Hispanic, non-Hispanic Black, and non-Hispanic White], original reason for Medicare entitlement [disabled], and Social Vulnerability Index [scored from 0 to 1]). The DID estimate of interest (ie, the indicator for intervention counties in post–policy year) captured the mean change in enrollment outcomes associated with the look-alike termination policy (ie, changes beyond the temporal changes of the control counties) (eMethods 1 in Supplement 1). We tested for differential prepolicy trends in both primary and secondary outcomes between intervention and control counties (eFigure 1 and eFigure 2A in Supplement 1). To address possible violations of the parallel trends assumption, we included a linear time trend and its interaction with the intervention county indicator given that observed differences in prepolicy trends were approximately linear (eFigure 2A and B in Supplement 1).^24^ All models were weighted by county-level, full-benefit, dual-eligible population, and robust SEs were clustered at the state level to accommodate potential nonindependence within states.

To understand to what extent the enrollment changes associated with the look-alike termination policy reflected the availability of different plan types in a given county, we repeated the DID analyses for each enrollment outcome, restricting the sample to counties where the corresponding plan type was available throughout the study period. This allowed us to isolate enrollment changes within existing plan offerings from changes driven by the introduction or absence of certain plan types following policy adoption.

In sensitivity analysis, we identified each look-alike plan’s predominant posttermination destination of beneficiaries and summarized the distribution of these destination plan types across terminated look-alike plans. We also repeated the DID analyses without adjusting for differential prepolicy trends. We conducted additional DID analyses separately limiting the intervention group to (1) counties with look-alike plans in 2022 and (2) counties with look-alike plans throughout the entire prepolicy period to ensure that changes in enrollment trends were not influenced by the entry or exit of look-alike plans prior to the policy. Finally, to examine potential dose-response effects, we further exploited variation in the prepolicy share of dual-eligible individuals enrolled in look-alike plans across counties to assess whether changes associated with the look-alike termination policy differed by the intensity of look-alike penetration, measured as the percentage of dual-eligible enrollment in look-alike plans between 2017 and 2022.

Analyses were performed using SAS version 9.4 (SAS Institute, Inc). The threshold for significance was 2-sided *P* < .05.

## Results

### Annual Trends in Number of Look-Alike Plans and Their Dual-Eligible Enrollment

**Figure 1.**
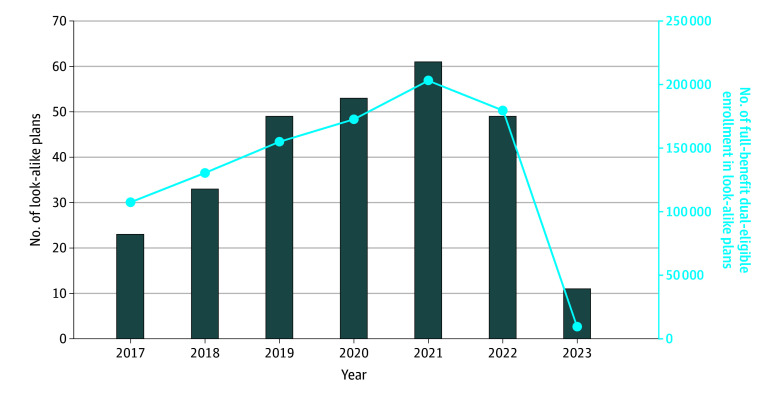
Annual Number of Look-Alike Plans and Their Dual-Eligible Enrollment, 2017–2023 Look-alike plans were identified based on January enrollment of full-benefit and partial-benefit dual-eligible beneficiaries in each year. Analysis was limited to plans offered in 50 states and Washington, DC. Special needs plans, employer plans, cost plans, and Medicare Savings Account plans were excluded from the analyses. Plans that were active for less than 1 year and with 200 or fewer total Medicare enrollees, as well as plans offered in states without dual-eligible special needs plans, were excluded from the analysis.

The number of look-alike plans increased steadily from 23 in 2017 to 61 in 2021, decreasing to 49 in 2022, before sharply dropping to 11 in 2023 (Figure 1). Full-benefit dual-eligible enrollment in look-alike plans increased from 107 421 in 2017 to a peak of 203 276 in 2021, slightly decreased to 179 581 in 2022, and then further decreased to 9550 in 2023 (Figure 1). The 11 plans identified as look-alikes in 2023 were newly classified as such and had not met the criteria in 2022 and therefore were not terminated.

### Beneficiary-Level Analysis of Enrollment Changes Following Look-Alike Plan Termination

**Figure 2.**
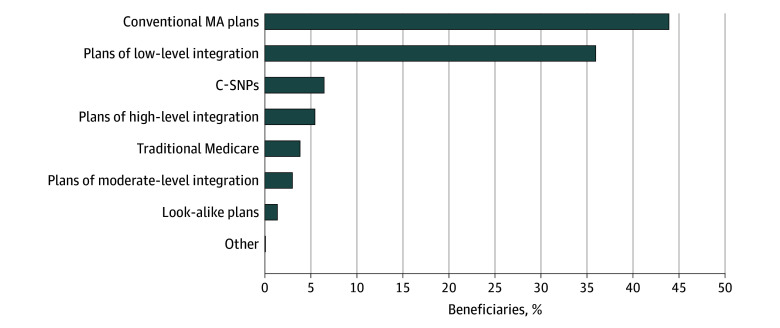
Transition of Full-Benefit Dual-Eligible Individuals on 2023 Centers for Medicare & Medicaid Services (CMS) Look-Alike Plan Termination Analysis was limited to full-benefit dual-eligible individuals enrolled in look-alike plans in 2022 who remained alive and eligible for full Medicaid benefits in 2023. Other includes institutional special needs plans and employer group waiver plans. Two MA plans had dual-eligible enrollment exceeding 80% as of January 2022 but were not classified as look-alike plans by CMS. Both of these plans continued to exceed the 80% threshold in January 2023 and were identified as look-alike plans in our analysis. California is the only state that has applicable integrated plan coordination-only dual-eligible special needs plans in 2023. These are classified as plans of low-level integration. C-SNP indicates chronic condition special needs plan; MA, Medicare Advantage.

Of the 170 399 full-benefit dual-eligible individuals enrolled in look-alike plans in 2022 who remained alive and dual-eligible in 2023 (58.9% female; 20.6% Asian, 44.8% Hispanic, 11.3% non-Hispanic Black, 21.4% non-Hispanic White, and 2% other), 5.4% transitioned into highly integrated plans, 39.0% moved into plans with moderate- or low-level integration, and the remaining 55.6% transitioned to nonintegrated plans—6.4% to C-SNPs, 0.06% to I-SNPs, 43.9% to conventional MA plans, 3.8% to traditional Medicare, and 1.4% to other look-alike plans—in 2023 (Figure 2). Compared with dual-eligible individuals enrolled in nonintegrated plans, those transitioning to highly integrated plans were more likely to be older (aged 65-74 years: adjusted differences: 3.4 percentage points [pp], 95% CI, 2.8-4.1 pp; aged 75-84 years: adjusted differences: 4.1 pp, 95% CI, 3.3-4.8 pp; aged ≥85 years: adjusted differences: 5.0 pp, 95% CI, 4.0-5.9 pp), female (adjusted difference: 0.6 pp, 95% CI, 0.2-0.9 pp), originally entitled to Medicare based on age rather than disability (adjusted difference: −0.7 pp, 95% CI, −1.2 to −0.2 pp), and to have higher HCC risk scores (quartile 2: adjusted differences: 1.2 pp, 95% CI, 0.7-1.7 pp; quartile 3: adjusted differences: 1.3 pp, 95% CI, 0.8-1.8 pp; quartile 4: adjusted differences: 1.1 pp, 95% CI, 0.6-1.6 pp). They were also significantly less likely to be Asian (adjusted difference: −5.0 pp, 95% CI, −5.6 to −4.4 pp) or non-Hispanic Black (adjusted difference: −0.9 pp, 95% CI, −1.6 to −0.2pp) (Table). In contrast, those enrolled in plans with lower levels of integration were more likely to be Asian (adjusted difference: 5.7 pp, 95% CI, 4.9-6.5pp), Hispanic (adjusted difference: 2.8 pp, 95% CI, 2.1-3.5 pp), and non-Hispanic Black (adjusted difference: 1.3 pp, 95% CI, 0.3-2.3 pp), and have lower HCC risk scores (quartile 3: adjusted differences: −0.9 pp, 95% CI, −1.5 to −0.2; quartile 4: adjusted differences: −1.1 pp, 95% CI, −1.8 to −0.5). In 40 of 49 look-alike plans enrolling full-benefit dual-eligible individuals in 2022, most dual-eligible beneficiaries transitioned to another plan offered by the same parent organization following the look-alike termination.

**Table.** Characteristics of Full-Benefit Dual-Eligible Individuals Transitioning Into Plans With Medicare-Medicaid Integration on Look-Alike Plan Termination, 2023^a^

Patient characteristic	Adjusted probability difference, % (95% CI)	
	Enrollment in plans with high-level integration	Enrollment in plans with low- or moderate-level integration
Sex		
Male	[Reference]	[Reference]
Female	0.6 (0.2 to 0.9)	0.0 (−0.5 to 0.4)
Age group		
<55 y	[Reference]	[Reference]
55-64 y	−1.6 (−2.2 to −0.9)	1.0 (−0.1 to 2.1)
65-74 y	3.4 (2.8 to 4.1)	2.8 (1.8 to 3.8)
75-84 y	4.1 (3.3 to 4.8)	4.5 (3.4 to 5.6)
≥85 y	5.0 (4.0 to 5.9)	3.2 (1.9 to 4.4)
Race and ethnicity^b^		
Asian	−5.0 (−5.6 to −4.4)	5.7 (4.9 to 6.5)
Hispanic	0.0 (−0.5 to 0.6)	2.8 (2.1 to 3.5)
Non-Hispanic Black	−0.9 (−1.6 to −0.2)	1.3 (0.3 to 2.3)
Non-Hispanic White	[Reference]	[Reference]
Other^c^	−1.1 (−2.6 to 0.3)	0.4 (−1.3 to 2.1)
Original reason for entitlement		
Old age and survivor’s insurance	[Reference]	[Reference]
DIB	−0.7 (−1.2 to −0.2)	0.4 (−0.3 to 1.0)
ESKD	−3.7 (−6.8 to −0.7)	−9.0 (−14.1 to −3.9)
DIB and ESKD	−4.3 (−8.0 to −0.7)	−10.6 (−17.6 to −3.6)
HCC risk score, quartile		
1 (Lowest)	[Reference]	[Reference]
2	1.2 (0.7 to 1.7)	1.0 (0.4 to 1.7)
3	1.3 (0.8 to 1.8)	−0.9 (−1.5 to −0.2)
4 (Highest)	1.1 (0.6 to 1.6)	−1.1 (−1.8 to −0.5)

Abbreviations: DIB, disability insurance benefits; ESKD, end-stage kidney disease; HCC, Hierarchical Condition Category.

^a^ Analysis was limited to full-benefit dual-eligible individuals enrolled in look-alike plans in 2022 who remained alive and eligible for full Medicaid benefits in 2023. More than 99% of these beneficiaries were enrolled in contracts with high Medicare Advantage encounter data completeness. The analysis compared the characteristics of full-benefit dual-eligible individuals transitioned from look-alike plans to integrated plans vs nonintegrated plans in 2023. For the model estimating the likelihood of enrollment in plans with high-level integration, the sample included full-benefit dual-eligible individuals transitioned from look-alike plans to plans with high-level integration and those moved to nonintegrated plans. For the model estimating the likelihood of enrollment in plans with low- or moderate-level integration, the sample included full-benefit dual-eligible individuals transitioned from look-alike plans to plans with low- or moderate-level integration and those who moved to nonintegrated plans. Adjusted probability differences were estimated using logistic regression models, where the dependent variable was enrollment into integrated plans (defined as plans with high or low/moderate level of integration, respectively), and explanatory variables included age, sex, race, original reason for Medicare entitlement, HCC risk score quartiles, and county fixed effects. California is the only state that has applicable integrated plan (AIP) coordination-only dual-eligible special needs plans (Co-D-SNPs) in 2023. These AIP Co-D-SNPs are classified as plans of low-level integration along with other Co-D-SNPs.

^b^ Race and ethnicity were defined using the Research Triangle Institute race code variable.^23^

^c^ Other includes American Indian, Alaska Native, other race or ethnicity categories that are not non-Hispanic White, Black, or Hispanic, as well as unknown.

### County-Year–Level DID Analysis

Between 2017 and 2022, 482 counties had full-benefit dual-eligible individuals enrolled in look-alike plans (ie, intervention counties), while 2094 counties did not have enrollment in plans meeting the CMS look-alike criteria (ie, control counties). Compared with control counties, intervention counties had a higher proportion of older and Hispanic dual-eligible individuals (for example, aged 75-84 years: 21.3% vs 17.1%; Hispanic ethnicity: 30.4% vs 13.7%). These counties were also more frequently located in the Midwest (43.6% vs 26.4%) or West (22.4% vs 8.1%) regions, and they were more likely to be urban (93.4% vs 81.8%) (eTable 1 in Supplement 1).

**Figure 3.**
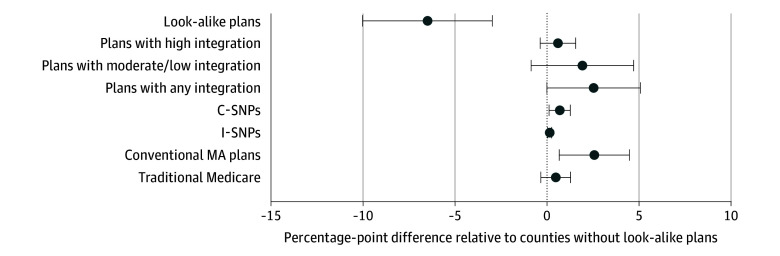
Adjusted Change in Dual-Eligible Enrollment Following 2023 Look-Alike Plan Termination Adjusted enrollment changes were estimated using a county-year–level difference-in-differences model that regressed each enrollment outcome on a post–policy year indicator, the indicator for intervention counties in post–policy year, county fixed effects, and time-varying county-level characteristics. Each dot represents the adjusted percentage point difference in dual-eligible enrollment between counties with and without look-alike plans following the 2023 look-alike plan termination policy. Further information on assumptions and the applicable integrated plan in California is available in eTable 2 and the caption of eFigure 3 in Supplement 1. Error bars represent the 95% CI. The vertical dashed line denotes no difference between counties with and without look-alike plans. C-SNP indicates chronic condition special needs plan; I-SNP, institutional special needs plan; MA, Medicare Advantage.

In DID analyses, the look-alike termination policy was not associated with any significant differential increase in dual-eligible enrollment into highly integrated plans comparing intervention counties with control counties (0.6 percentage points [pp]; 95% CI, −0.4 to 1.6 pp) (Figure 3). We found a 2.6-pp (95% CI, 0.01-5.1 pp) increase in enrollment into plans with any level of integration in intervention counties relative to control counties following the termination policy (Figure 3). This change was mainly driven by increased enrollment in plans with lower levels of integration in intervention counties relative to control counties post–policy implementation.

Following the implementation of the look-alike termination policy, there was a 2.6-pp increase (95% CI, 0.7-4.5 pp) in enrollment in conventional MA plans with dual-eligible enrollment less than 80% (Figure 3). Dual-eligible enrollment also increased by 0.7 pp (95% CI, 0.1-1.3 pp) in C-SNPs and by 0.16 pp (95% CI, 0.05-0.27 pp) in I-SNPs in intervention counties compared with control counties (Figure 3). We did not find any significant difference in enrollment changes for traditional Medicare between intervention and control counties (0.5 pp; 95% CI, −0.3 to 1.3 pp) (Figure 3).

Results remained largely consistent when we restricted the analysis to counties where each plan type of interest was consistently available throughout the study period. Specifically, the look-alike termination policy was not associated with any significant differential increase in enrollment into highly integrated plans in intervention vs control counties (1.0 pp; 95% CI, −0.3 to 2.3 pp), and it was associated with a 3.0-pp increase (95% CI, 0.9-5.1 pp) in enrollment into plans with a lower level of integration in intervention counties relative to control counties (eFigure 3 in Supplement 1).

In sensitivity analyses, for each look-alike plan active in 2022, we characterized the type of plan into which most full-benefit dual-eligible enrollees transitioned following termination in 2023. In just 1 of 49 terminated look-alike plans did the majority (58.3%) of its full-benefit dual-eligible enrollees move to a plan with a high-level of integration, while in 34 plans, the plurality of full-benefit dual-eligible individuals transitioned into plans with no Medicare-Medicaid integration (eFigure 4 in Supplement 1). The DID results were largely unchanged when models excluded adjustment for differential prepolicy trends (eFigure 5 in Supplement 1), although the increase in enrollment into plans with any Medicaid integration was no longer significant, reflecting slower prepolicy growth in such enrollment among intervention counties. Results also remained largely consistent when the intervention group was limited to counties with look-alike plans in 2022 (eFigure 6 in Supplement 1) or counties with look-alike plans throughout the prepolicy period (eFigure 7 in Supplement 1). We obtained similar findings when examining policy effects by the prepolicy share of dual-eligible individuals enrolled in look-alike plans (eFigure 8 in Supplement 1).

## Discussion

In this national study, the CMS policy to terminate D-SNP look-alike plans was not associated with increased enrollment into highly integrated care plans among full-benefit dual-eligible beneficiaries. In fact, only 5.4% of full-benefit dual-eligible individuals previously enrolled in look-alike plans transitioned into plans offering a high-level of integration, whereas more than half of enrollees in terminated plans transitioned into plans without any Medicare-Medicaid integration. Although enrollment into plans with any form of integration increased after accounting for prepolicy trends, this trend was primarily driven by enrollment in plans with lower levels of integration. Additionally, we observed relative increases in enrollment into other nonintegrated plans not subject to the look-alike termination policy, including C-SNPs, I-SNPs, and conventional MA plans with fewer than 80% dual-eligible enrollees.

Taken together, these results suggest that the look-alike termination policy did not achieve its goal of expanding dual-eligible enrollment into plans with an adequate level of Medicare-Medicaid integration. While it may be encouraging to observe increases in dual-eligible enrollment in plans with low-level integration, specifically Co-D-SNPs, these plans assume no financial responsibility for Medicaid services and only offer limited care coordination.^4^ Consequently, beneficiaries in these plans must continue navigating 2 separate systems. In contrast, highly integrated plans manage and finance both Medicare and Medicaid services under 1 entity, potentially enabling more coordinated care while reducing incentives to shift costs between programs.^4^ Prior research has shown that Co-D-SNPs did not significantly improve patient outcomes compared with conventional MA plans, whereas highly integrated plans such as fully integrated dual-eligible SNPs were associated with better outcomes in some areas, such as enrollee satisfaction and providing long-term services and supports in community rather than institutional settings.^5,25^

Of note, enrollment increased in conventional MA plans with dual-eligible enrollment below the 80% threshold following the look-alike termination policy. This raises a concern that insurers may have shifted dual-eligible individuals from terminated plans to other nonintegrated MA plans with dual-eligible enrollment below the 80% threshold, instead of moving enrollees into integrated care plans.^7^ Although CMS will further lower the threshold for classifying look-alike plans in upcoming years,^12^ our findings underscore considerable uncertainty in whether the revised thresholds will substantially increase enrollment in integrated care plans.

Additionally, we observed a modest but significant increase in dual-eligible enrollment into C-SNPs and I-SNPs. While C-SNPs provide tailored benefits for individuals with chronic conditions and I-SNPs deliver specialized care for those requiring long-term services, neither of these plan types was subject to requirements to coordinate Medicaid services or manage Medicaid spending. Because such plans are exempted from the look-alike termination policy,^11^ they may serve as a regulatory workaround for MA plans seeking to maintain dual-eligible enrollment. Prior research estimated that over 10% of C-SNPs would have met look-alike criteria had the policy applied to them.^22^ Continued growth of C-SNPs, which served over 1 million beneficiaries in 2025,^26^ could further undermine federal integrated care efforts.

As policymakers seek to increase dual-eligible enrollment in MA plans with high Medicare-Medicaid integration, efforts could focus on 3 fronts. First, states and CMS could consider default enrollment of dual-eligible individuals from terminated plans into an insurer’s integrated care plan, when one is available. Second, policymakers could extend look-alike restrictions to I-SNPs and C-SNPs with high shares of dual-eligible enrollees, or alternatively, require a minimum level of Medicaid integration currently applicable to coordination-only D-SNPs. Third, policymakers could provide further state support, oversight, and resources to help expand access to highly integrated plan options in areas with limited availability.

### Limitations

This study had several limitations. First, although we controlled for time-varying county-level characteristics and adjusted for potential differences in dual-eligible enrollment trends prior to the termination policy, there may still be unmeasured time-varying differences between counties with and without look-alike plans that could bias our estimates. Second, by using counties without look-alike plans as controls, our estimates reflect only differential changes between intervention and control counties; any potential spillover effects extending to counties without look-alike plans are not captured. Third, since CMS announced its intent to terminate look-alike plans in 2020, MA plans may have begun adjusting their enrollment strategies prior to the 2023 policy implementation, potentially undermining the parallel trends assumption and biasing effect estimates. Fourth, we did not separately assess enrollment in plans with moderate- and low-level integration, as highly integrated D-SNPs were not formally designated until 2021, limiting preintervention data for this outcome.

## Conclusions

This national study found no evidence that the CMS look-alike termination policy was associated with increased enrollment in highly integrated plans. While there was a modest increase in enrollment into plans with lower levels of integration, concerns remain that most dual-eligible individuals remain in plans that do not adequately financially integrate Medicare and Medicaid services. These findings suggest that the current CMS policy to terminate look-alike plans alone may be insufficient to promote enrollment in plans attaining high Medicare-Medicaid integration.

## Notes

Supplement 1. **eMethods 1**. Regression Specification **eFigure 1**. Adjusted Annual Changes in Dual-eligible Enrollment in Highly Integrated Plans Before and After the Look-alike Plan Termination Policy, 2017-2023 **eFigure 2**A. Unadjusted Full-Benefit Dual-eligible Enrollment Trend by Plan Type, 2017-2023 **eFigure 2**B. Adjusted Full-Benefit Dual-eligible Enrollment Trend by Plan Type, 2017-2023 **eTable 1.** Characteristics of Counties With and Without Look-alike Plans, 2022 **eFigure 3**. Adjusted Change in Dual-eligible Enrollment Following 2023 Look-alike Plan Termination, Limited to Counties with Plan Type Available Throughout 2017-2023 **eFigure 4**. 2023 Most Common Plan Destination of 2022 Look-Alike Plans for Full-Benefit Dual-eligibles **eFigure 5**. Adjusted Change in Dual-eligible Enrollment Following 2023 Look-alike Plan Termination, No Adjustment for Pre-Policy Differential Trends **eFigure 6**. Adjusted Change in Dual-eligible Enrollment Following 2023 Look-alike Plan Termination, Intervention Counties Limited to Those with Look-alike Plans in 2022 **eFigure 7**. Adjusted Change in Dual-eligible Enrollment Following 2023 Look-alike Plan Termination, Intervention Counties Limited to Those with Look-alike Plans Throughout 2017-2022 **eFigure 8**. Adjusted Change in Dual-eligible Enrollment Following 2023 Look-alike Plan Termination, Per Additional Percentage Point Dual-eligible Look-alike Enrollment at Baseline **eTable 2.** List of California Co-D-SNPs Identified as Receiving MMP Enrollment in January 2023

Supplement 2.
Data Sharing Statement
