# Supplementary material for: Federal Look-Alike Plan Termination Policy and Dual-Eligible Enrollment in Integrated Care Programs
Source: JAMA Health Forum. 2026 Jan 16;7(1):e256294. doi: 10.1001/jamahealthforum.2025.6294 (PMC12811809; doi:10.1001/jamahealthforum.2025.6294)
Supplement: Supplement 1. — eMethods 1. Regression Specification eFigure 1. Adjusted Annual Changes in Dual-eligible Enrollment in Highly Integrated Plans Before and After the Look-alike Plan Termination Policy, 2017-2023 eFigure 2A. Unadjusted Full-Benefit Dual-eligible Enrollment Trend by Plan Type, 2017-2023 eFigure 2B. Adjusted Full-Benefit Dual-eligible Enrollment Trend by Plan Type, 2017-2023 eTable 1. Characteristics of Counties With and Without Look-alike Plans, 2022 eFigure 3. Adjusted Change in Dual-eligible Enrollment Following 2023 Look-alike Plan Termination, Limited to Counties with Plan Type Available Throughout 2017-2023 eFigure 4. 2023 Most Common Plan Destination of 2022 Look-Alike Plans for Full-Benefit Dual-eligibles eFigure 5. Adjusted Change in Dual-eligible Enrollment Following 2023 Look-alike Plan Termination, No Adjustment for Pre-Policy Differential Trends eFigure 6. Adjusted Change in Dual-eligible Enrollment Following 2023 Look-alike Plan Termination, Intervention Counties Limited to Those with Look-alike Plans in 2022 eFigure 7. Adjusted Change in Dual-eligible Enrollment Following 2023 Look-alike Plan Termination, Intervention Counties Limited to Those with Look-alike Plans Throughout 2017-2022 eFigure 8. Adjusted Change in Dual-eligible Enrollment Following 2023 Look-alike Plan Termination, Per Additional Percentage Point Dual-eligible Look-alike Enrollment at Baseline eTable 2. List of California Co-D-SNPs Identified as Receiving MMP Enrollment in January 2023 [file jamahealthforum-e256294-s001.pdf]

Supplemental Online Content

Ma Y, Roberts ET, Phelan J, et al. Federal look-alike plan termination policy and dual-eligible enrollment in integrated care programs. *JAMA Health Forum*. 2026;7(1):e256294. doi:10.1001/jamahealthforum.2025.6294

eMethods 1. Regression Specification

eFigure 1. Adjusted Annual Changes in Dual-eligible Enrollment in Highly Integrated Plans Before and After the Look-alike Plan Termination Policy, 2017-2023

eFigure 2A. Unadjusted Full-Benefit Dual-eligible Enrollment Trend by Plan Type, 2017-2023

eFigure 2B. Adjusted Full-Benefit Dual-eligible Enrollment Trend by Plan Type, 2017-2023

eTable 1. Characteristics of Counties With and Without Look-alike Plans, 2022

eFigure 3. Adjusted Change in Dual-eligible Enrollment Following 2023 Look-alike Plan Termination, Limited to Counties with Plan Type Available Throughout 2017-2023

eFigure 4. 2023 Most Common Plan Destination of 2022 Look-Alike Plans for Full-Benefit Dual-eligibles

eFigure 5. Adjusted Change in Dual-eligible Enrollment Following 2023 Look-alike Plan Termination, No Adjustment for Pre-Policy Differential Trends

eFigure 6. Adjusted Change in Dual-eligible Enrollment Following 2023 Look-alike Plan Termination, Intervention Counties Limited to Those with Look-alike Plans in 2022

eFigure 7. Adjusted Change in Dual-eligible Enrollment Following 2023 Look-alike Plan Termination, Intervention Counties Limited to Those with Look-alike Plans Throughout 2017-2022

eFigure 8. Adjusted Change in Dual-eligible Enrollment Following 2023 Look-alike Plan Termination, Per Additional Percentage Point Dual-eligible Look-alike Enrollment at Baseline

eTable 2. List of California Co-D-SNPs Identified as Receiving MMP Enrollment in January 2023

This supplemental material has been provided by the authors to give readers additional information about their work.

© 2026 Ma Y et al. *JAMA Health Forum*.

eMethods 1 Regression Specification

A. Difference-in-differences Analyses without Pre-trend Adjustment

We estimated the following difference-in-differences regression to compare changes in full-benefit dual-eligible enrollment at the county-year level before (2017-2022) versus after implementation of the look-alike termination policy (2023) between counties with and without look-alike plans:

$$Y_{st} = \beta_0 + \beta_1 \mathrm{Post}_\mathrm{t} + \beta_2 \mathrm{LookAlike}_\mathrm{s} * \mathrm{Post}_\mathrm{t} + \beta_3' X_{st} + \theta_s + \epsilon_{st}$$

where s denotes county and t denotes year. Y_{st} denotes the outcome of interest, i.e., proportion of full-benefit dual-eligibles enrolled in a given plan type in county s and year t. $LookAlike_s$ denotes whether county s had look-alike plans in pre-policy period. Post_t denotes whether look-alike termination policy is adopted in year t. X_{st} denotes a vector of time-varying county-level demographical variables. θ_s denotes county fixed effects. The coefficient β_2 of the interaction term $\mathrm{LookAlike}_\mathrm{s} * \mathrm{Post}_\mathrm{t}$ captures the average change in full-benefit dual-eligible enrollment in a given plan type associated with the implementation of the look-alike termination policy.

B. Difference-in-differences Analyses with Pre-trend Adjustment

Given the potential violations of the parallel trend assumption for secondary outcomes, we also estimated the following difference-in-differences design adjusting for differential pre-policy enrollment trends between counties with and without look-alike plans:

$$Y_{st} = \beta_0 + \beta_1 \mathrm{Post}_\mathrm{t} + \beta_2 \mathrm{LookAlike}_\mathrm{s} * \mathrm{Post}_\mathrm{t} +$$

$$\beta_3 \mathrm{T} + \beta_4 \mathrm{T} * \mathrm{LookAlike}_\mathrm{s} + \beta_5' X_{st} + \theta_s + \epsilon_{st}$$

© 2026 Ma Y et al. *JAMA Health Forum*.

where T represents a linear year term. The interaction $\mathrm{T} * \mathrm{LookAlike_s}$ captures any potential difference in linear year trends between counties with and without look-alike plans. The coefficient β_2 of the interaction term $\mathrm{LookAlike_s} * \mathrm{Post_t}$ captures the average change in full-benefit dual-eligible enrollment in a given plan type associated with the implementation of the look-alike termination policy after adjusting for potential differential pre-policy enrollment trends between counties with and without look-alike plans.

© 2026 Ma Y et al. *JAMA Health Forum*.

eFigure 1 Adjusted Annual Changes in Dual-eligible Enrollment in Highly Integrated Plans Before and After the Look-alike Plan Termination Policy, 2017-2023

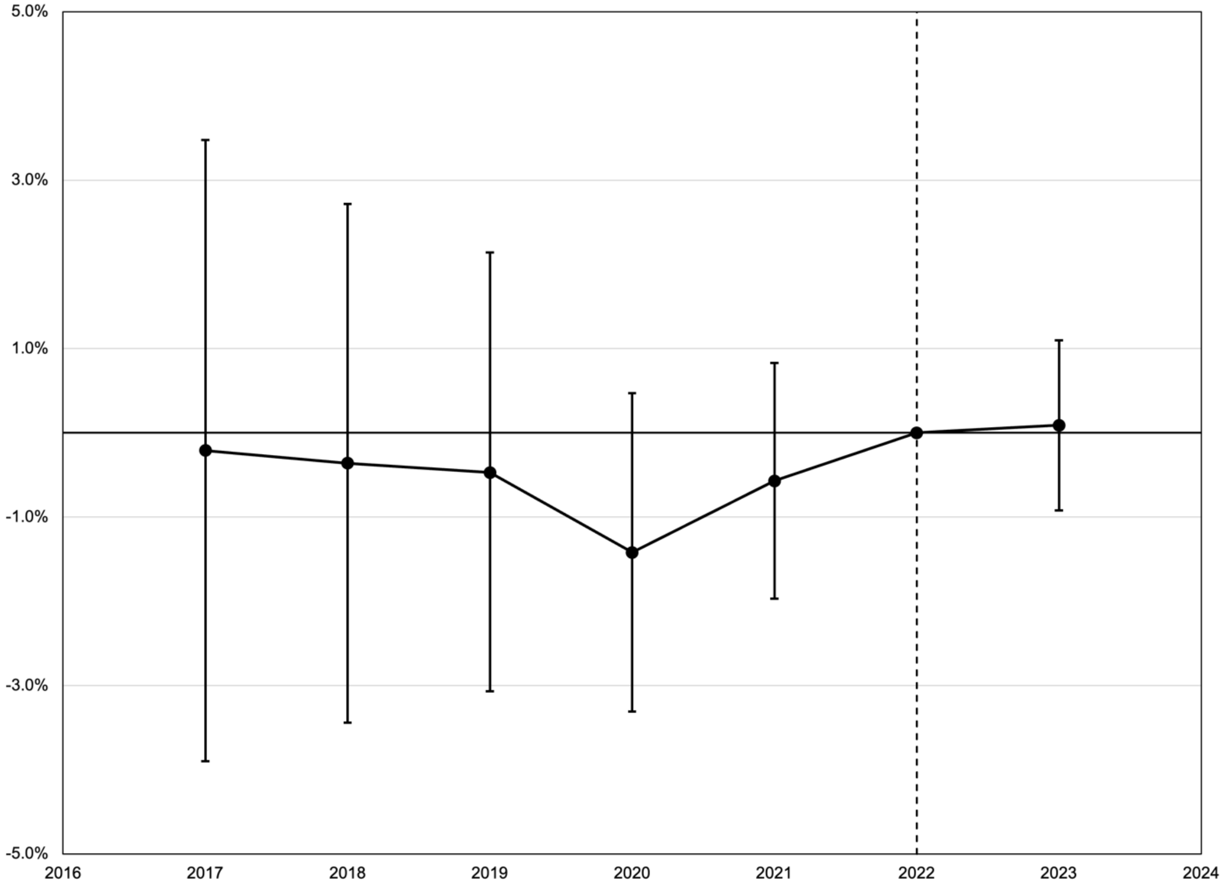

Notes:

1. Each circle represents the estimated change in full-benefit dual-eligible enrollment in highly integrated plans in counties with look-alike plans relative to counties without look-alike plans in the year compared to 2022. The estimates are adjusted for year fixed effects, county fixed effects, and time-varying county-level full-benefit dual-eligible characteristics. Error bar represents 95% confidence interval.

© 2026 Ma Y et al. *JAMA Health Forum*.

eFigure 2A Unadjusted Full-Benefit Dual-eligible Enrollment Trend by Plan Type, 2017-2023

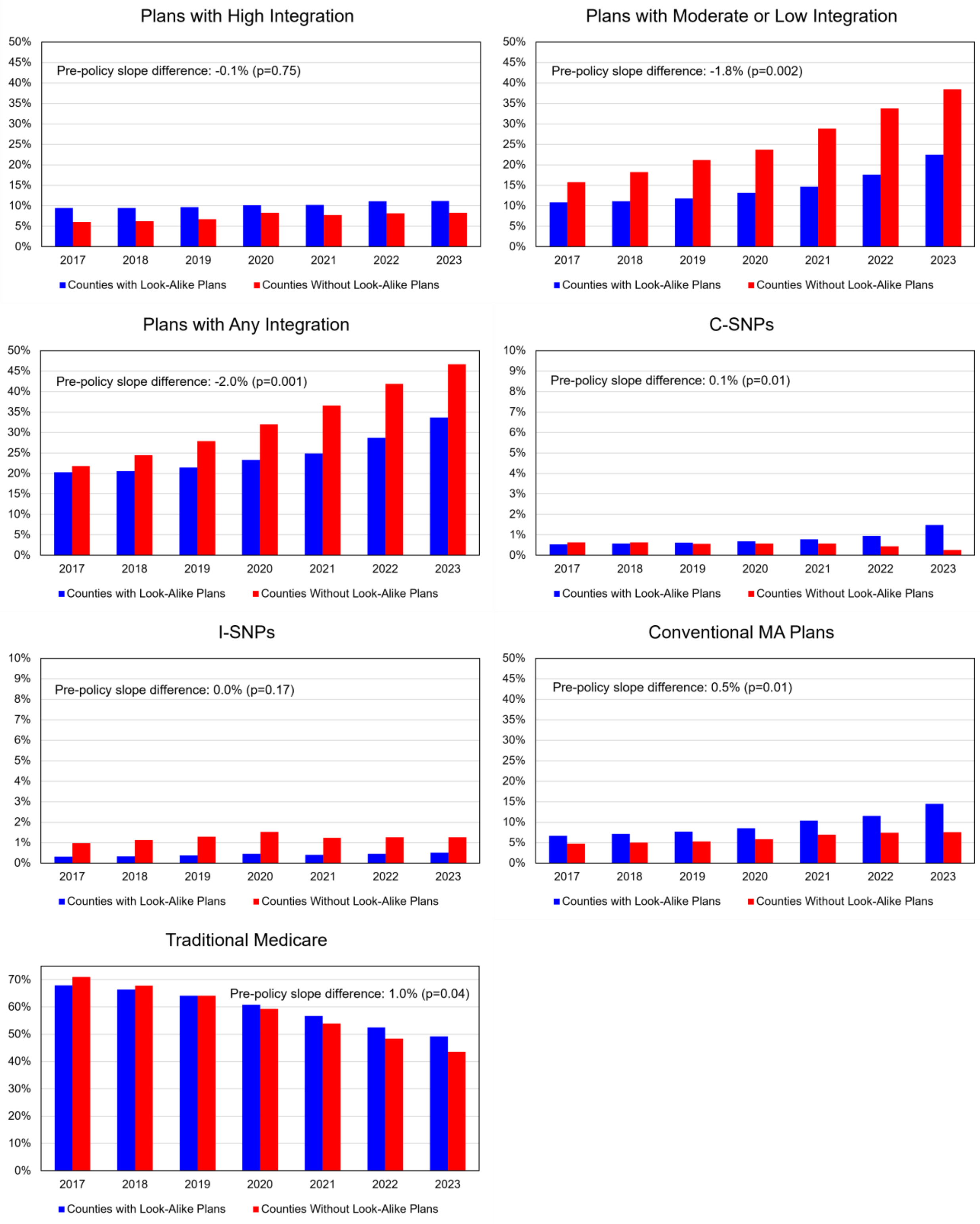

© 2026 Ma Y et al. *JAMA Health Forum*.

Notes:

1. Each bar represents the unadjusted proportion of full-benefit dual-eligibles enrolled in the corresponding plan type.
2. The pre-policy slope difference was estimated by regressing each outcome on a linear year term, its interaction with the intervention county indicator, county fixed effects, and time-varying county-level characteristics using data from 2017–2022. The coefficient of the interaction term represents the difference in pre-policy slopes between intervention and control counties.

© 2026 Ma Y et al. *JAMA Health Forum*.

eFigure 2B Adjusted Full-Benefit Dual-eligible Enrollment Trend by Plan Type, 2017-2023

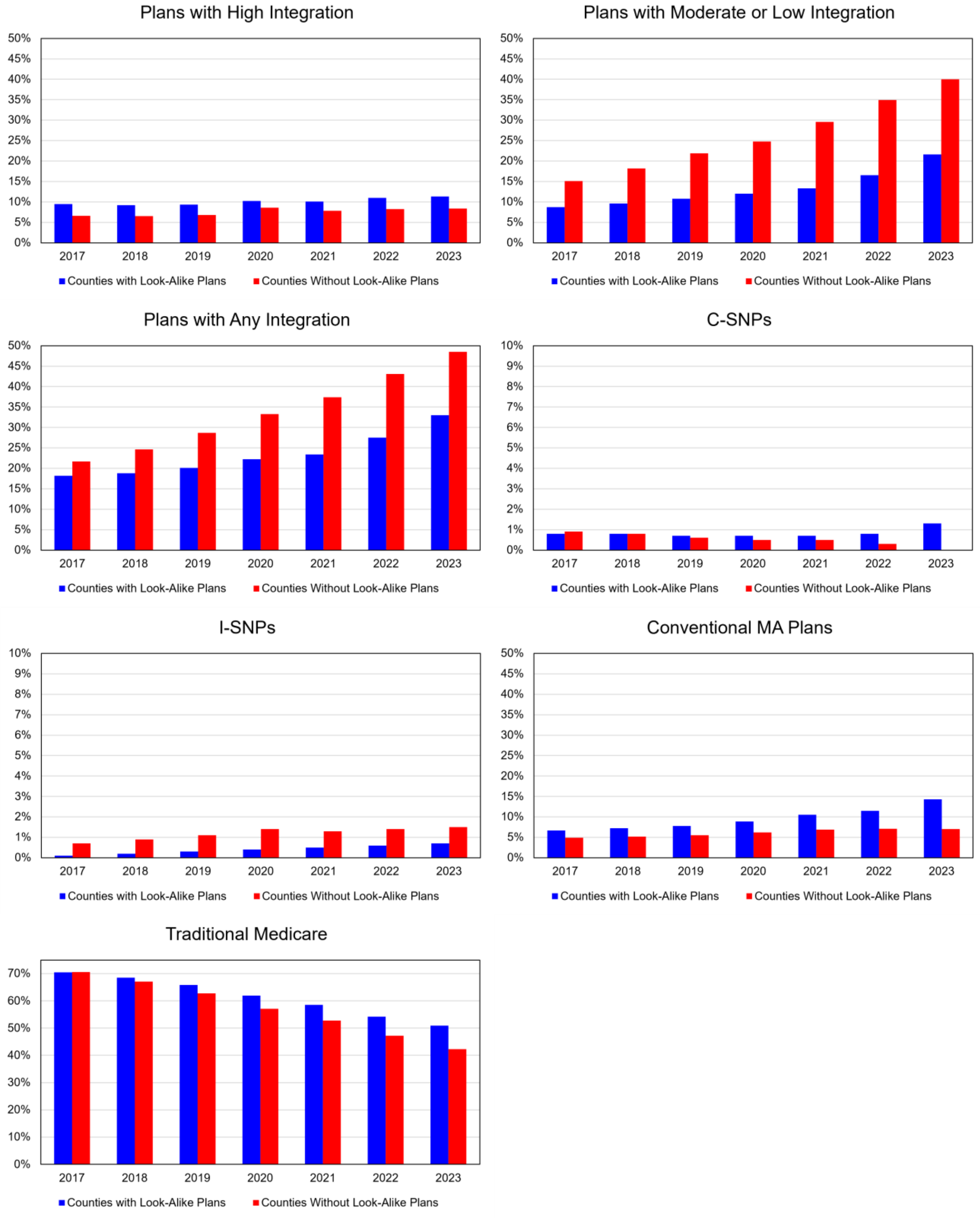

Notes:

© 2026 Ma Y et al. *JAMA Health Forum*.

1. Each bar represents the adjusted proportion of full-benefit dual-eligibles enrolled in the corresponding plan type.
2. Adjusted estimates were estimated using a county-year-level regression model that regressed each enrollment outcome on a binary indicator for intervention counties, year fixed effects, an interaction between intervention county and year fixed effects, and time-varying county-level characteristics.

© 2026 Ma Y et al. *JAMA Health Forum*.

eTable 1 Characteristics of Counties With and Without Look-alike Plans, 2022

	Counties with Look-Alike Plans	Counties Without Look-Alike Plans
Number of Counties	482	2,094
Number of Full-Benefit Dual-Eligibles	3,168,367	5,138,365
Full-Benefit Dual-Eligible Characteristics		
Age Group		
<65	30.2%	39.9%
65-74	36.7%	33.0%
75-84	21.3%	17.1%
85+	11.7%	10.0%
Sex		
Male	41.5%	40.8%
Female	58.5%	59.2%
Race/Ethnicity		
NH White	38.6%	52.6%
NH Black	15.0%	23.6%
Hispanic	30.4%	13.7%
Other	13.8%	7.9%
Unknown	2.2%	2.3%
Original Reason for Medicare Entitlement		
Old Age and Survivors Insurance	55.2%	44.1%
Disability Insurance Benefits (DIB)	43.7%	54.6%
End-Stage Renal Disease (ESRD)	0.7%	0.9%
Both DIB and ESRD	0.3%	0.4%
Medicare Coverage Type		
Traditional Medicare	51.0%	46.6%
Medicare Advantage	49.0%	53.4%
County-Level Characteristics		
Region		
Midwest	43.6%	26.4%
Northeast	3.7%	8.4%
South	30.3%	57.2%
West	22.4%	8.1%
Rurality		
Urban/Suburban	93.4%	81.8%
Rural	6.6%	18.2%
Average Social Vulnerability Index Score	0.524	0.529

Notes:

1. Rurality defined based on Rural-Urban Continuum Codes (RUCC).
2. Connecticut is excluded from the calculation of average Social Vulnerability Index score due to inconsistency of county definition during the study period.

© 2026 Ma Y et al. *JAMA Health Forum*.

eFigure 3 Adjusted Change in Dual-eligible Enrollment Following 2023 Look-alike Plan Termination, Limited to Counties with Plan Type Available Throughout 2017-2023

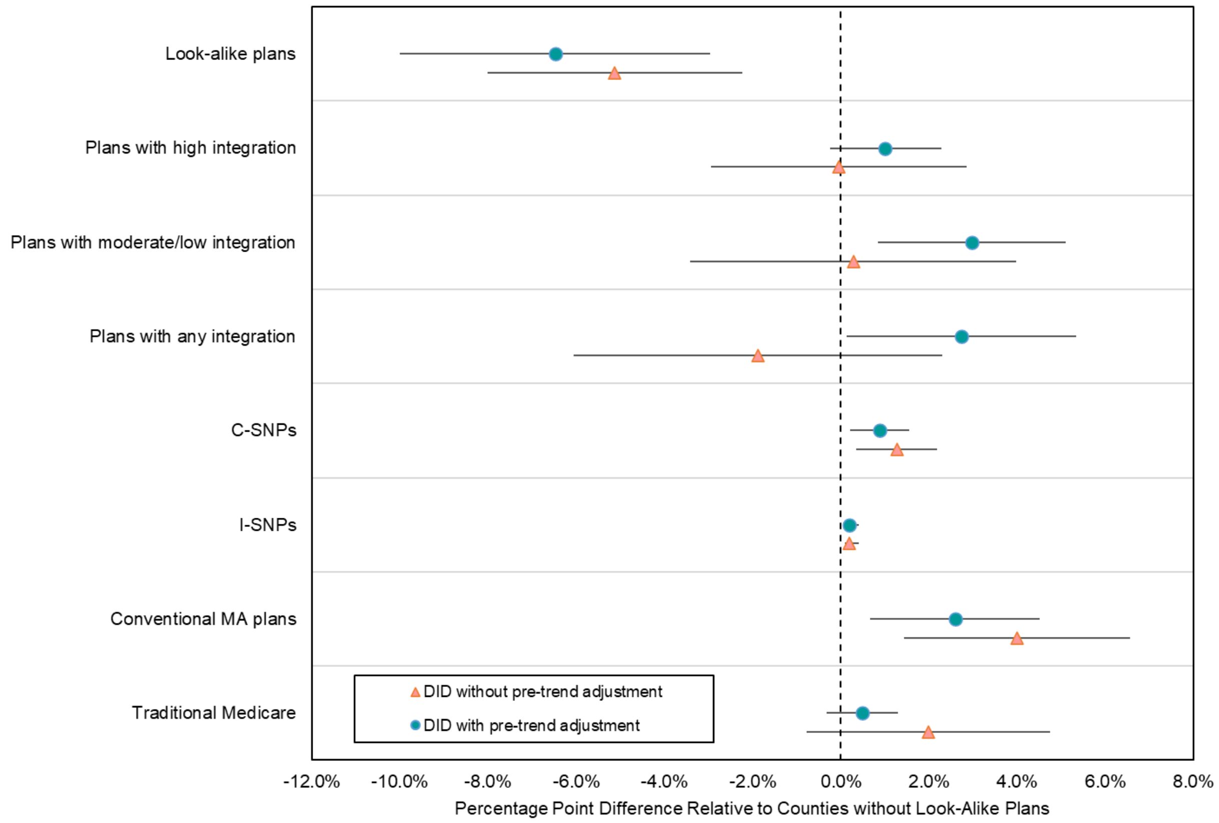

Notes:

1. For each plan type, analysis is limited to only counties with the corresponding plan type available throughout the study period.
2. Adjusted enrollment changes were estimated using a county-year-level difference-in-differences model that regressed each enrollment outcome on a post-policy year indicator, an indicator for intervention counties in post-policy year, county fixed effects, and time-varying county-level characteristics.
3. To address potential violations of the parallel trends assumption, both standard difference-in-differences estimates and estimates adjusting for differential pre-policy trends between intervention and control counties were reported. See eFigure 1 and eFigure 2 for annual enrollment outcome trends in intervention and control counties.
4. California is the only state that has Applicable Integrated Plan (AIP) Co-D-SNPs in 2023. California's MMP demonstration ended on December 31, 2022 and beneficiaries were transitioned to D-SNPs as of January 1, 2023. Among California AIP Co-D-SNPs, 7 were identified via the CMS Plan Crosswalk as receiving MMP enrollment in 2023 and were thus classified as plans with high-level integration. See eTable 2 for list of D-SNPs identified as receiving MMP enrollment.

© 2026 Ma Y et al. *JAMA Health Forum*.

eFigure 4 2023 Most Common Plan Destination of 2022 Look-Alike Plans for Full-Benefit Dual-eligibles

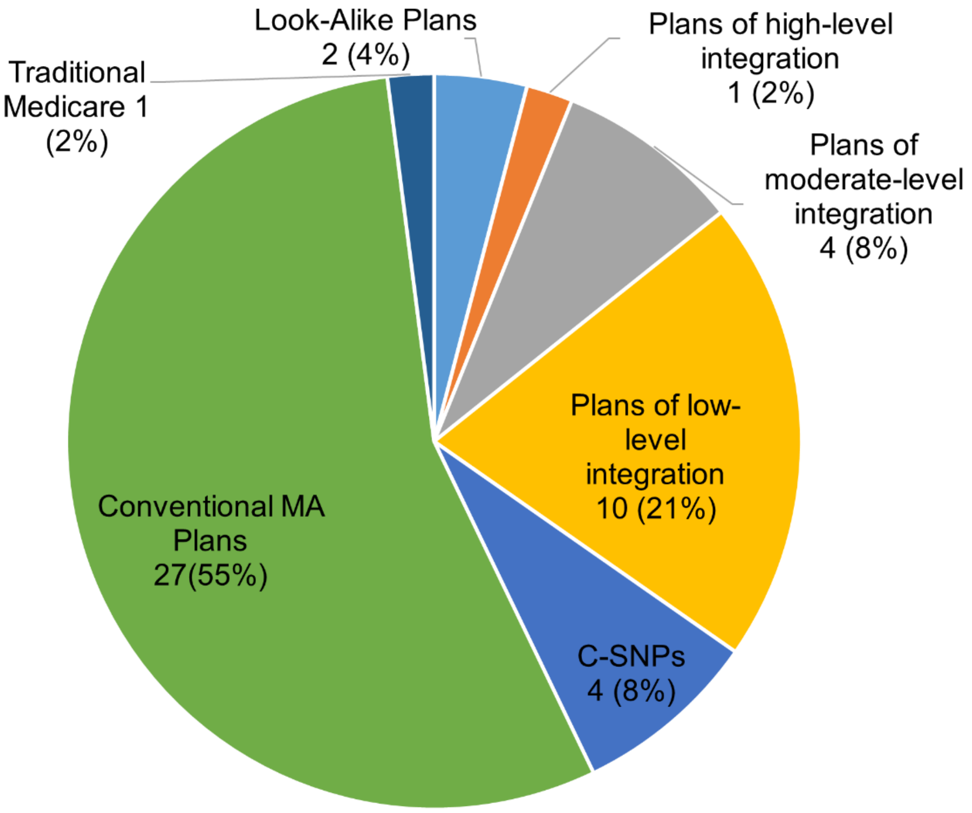

Notes:

1. Figure represents the number and proportion of 2022 look-alike plans in which full-benefit dual-eligibles most commonly transitioned into each plan type in 2023.
2. Two MA plans had dual-eligible enrollment exceeding 80% as of January 2022 but were not classified as look-alike plans by CMS. Both of these plans continued to exceed 80% threshold in January 2023 and were identified as look-alike plans in our analysis.
3. California is the only state that has Applicable Integrated Plan (AIP) Co-D-SNPs in 2023. These AIP Co-D-SNPs are classified as "plans of low-level integration" along with other Co-D-SNPs.

© 2026 Ma Y et al. *JAMA Health Forum*.

eFigure 5 Adjusted Change in Dual-eligible Enrollment Following 2023 Look-alike Plan Termination, No Adjustment for Pre-Policy Differential Trends

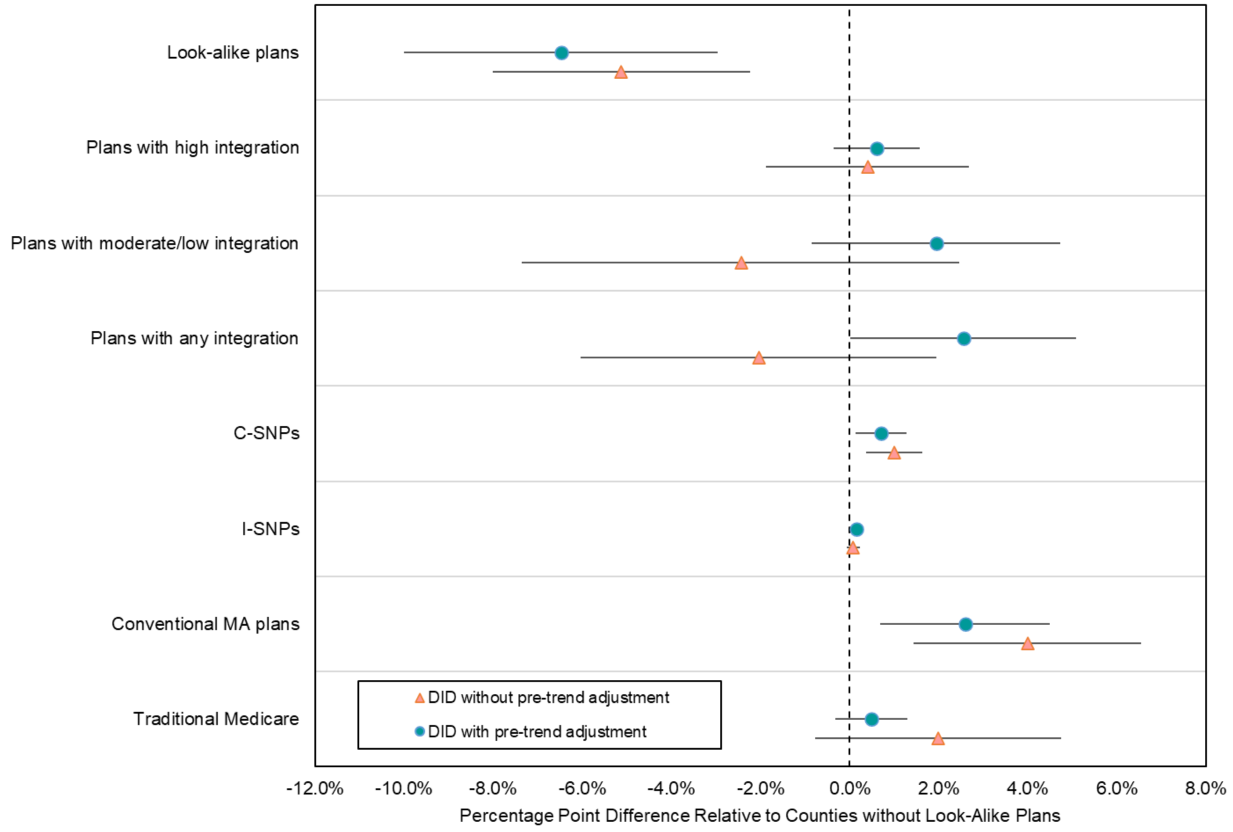

Notes:

1. Adjusted enrollment changes were estimated using a county-year-level difference-in-differences model that regressed each enrollment outcome on a post-policy year indicator, an indicator for intervention counties in post-policy year, county fixed effects, and time-varying county-level characteristics.
2. To address potential violations of the parallel trends assumption, both standard difference-in-differences estimates and estimates adjusting for differential pre-policy trends between intervention and control counties were reported.
3. California is the only state that has Applicable Integrated Plan (AIP) Co-D-SNPs in 2023. California's MMP demonstration ended on December 31, 2022 and beneficiaries were transitioned to D-SNPs as of January 1, 2023. Among California AIP Co-D-SNPs, 7 were identified via the CMS Plan Crosswalk as receiving MMP enrollment in 2023 and were thus classified as plans with high-level integration. See eTable 2 for list of D-SNPs identified as receiving MMP enrollment.

© 2026 Ma Y et al. *JAMA Health Forum*.

eFigure 6 Adjusted Change in Dual-eligible Enrollment Following 2023 Look-alike Plan Termination, Intervention Counties Limited to Those with Look-alike Plans in 2022

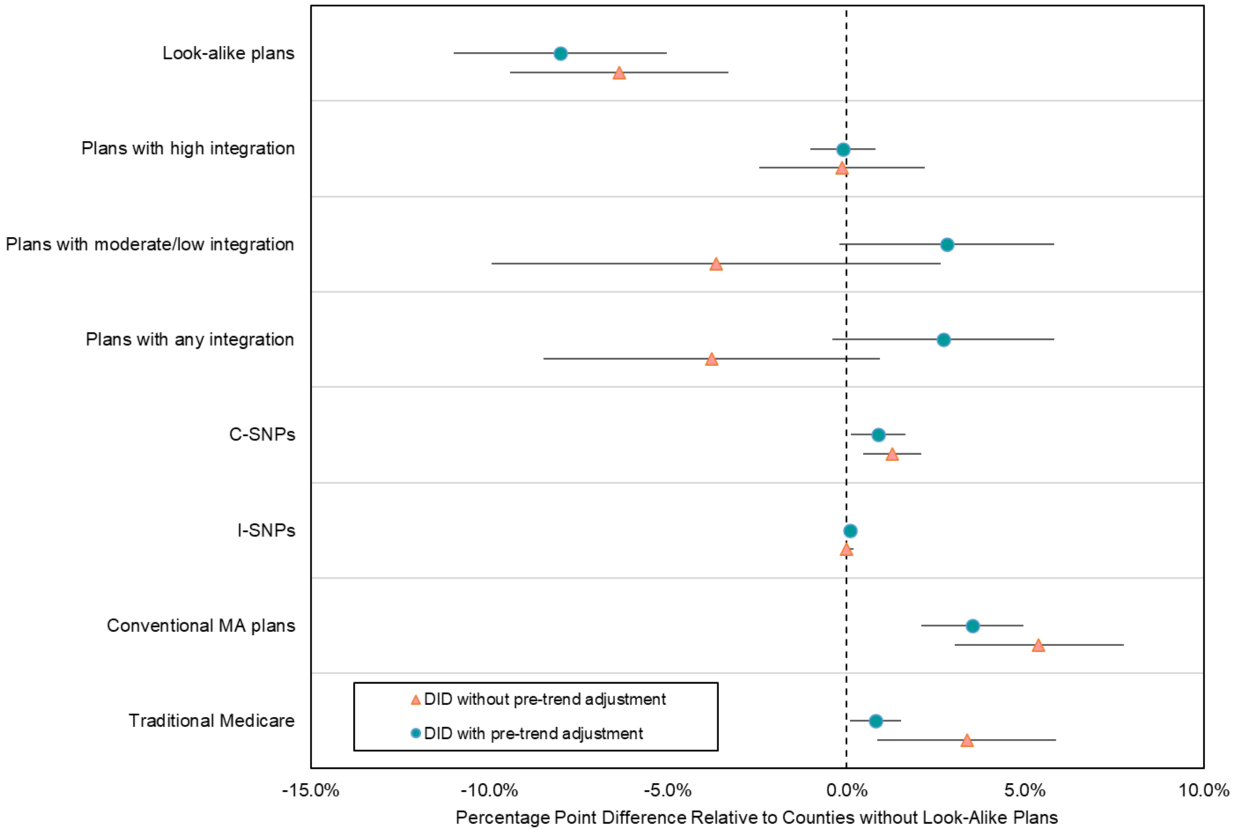

Notes:

1. Intervention counties are limited to 206 counties that had look-alike plans in 2022, i.e., the year right before the implementation of the look-alike termination policy. There were 2,451,593 full-benefit dual-eligibles residing in these counties in 2022, representing 77.4% of the full-benefit dual-eligible population that resided in counties ever had look-alike plans between 2017 and 2022.
2. Adjusted enrollment changes were estimated using a county-year-level difference-in-differences model that regressed each enrollment outcome on a post-policy year indicator, an indicator for intervention counties in post-policy year, county fixed effects, and time-varying county-level characteristics.
3. To address potential violations of the parallel trends assumption, both standard difference-in-differences estimates and estimates adjusting for differential pre-policy trends between intervention and control counties were reported. See eFigure 1 for annual enrollment outcome trends in intervention and control counties.
4. California is the only state that has Applicable Integrated Plan (AIP) Co-D-SNPs in 2023. California's MMP demonstration ended on December 31, 2022 and beneficiaries were transitioned to D-SNPs as of January 1, 2023. Among California AIP Co-D-SNPs, 7 were identified via the CMS Plan Crosswalk as receiving MMP enrollment in 2023 and were thus classified as plans with high-level integration. See eTable 2 for list of D-SNPs identified as receiving MMP enrollment.

© 2026 Ma Y et al. *JAMA Health Forum*.

eFigure 7 Adjusted Change in Dual-eligible Enrollment Following 2023 Look-alike Plan Termination, Intervention Counties Limited to Those with Look-alike Plans Throughout 2017-2022

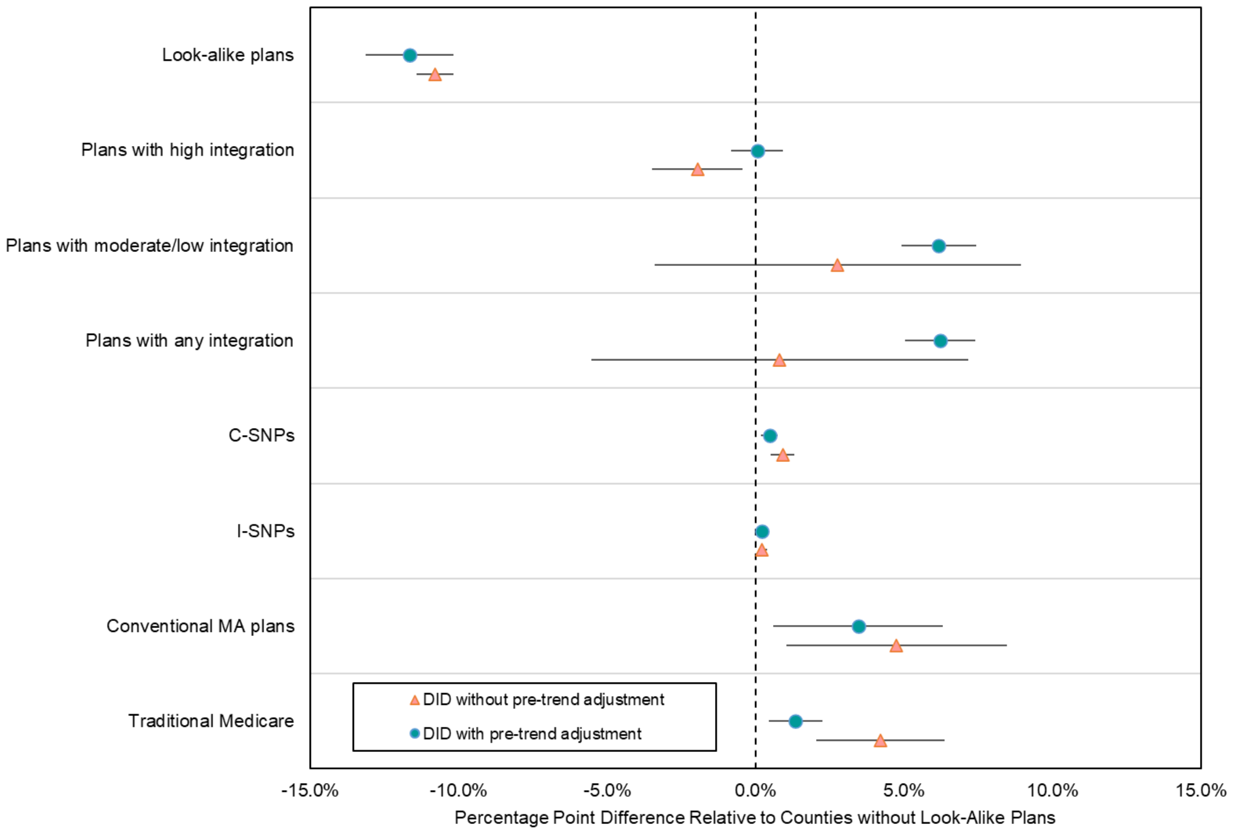

Notes:

1. Intervention counties are limited to 15 counties that consistently had look-alike plans in each year before the implementation of the look-alike termination policy. There were 1,288,123 full-benefit dual-eligibles residing in these counties in 2022, representing 40.7% of the full-benefit dual-eligible population that resided in counties that ever had look-alike plans between 2017 and 2022.
2. Adjusted enrollment changes were estimated using a county-year-level difference-in-differences model that regressed each enrollment outcome on a post-policy year indicator, an indicator for intervention counties in post-policy year, county fixed effects, and time-varying county-level characteristics.
3. To address potential violations of the parallel trends assumption, both standard difference-in-differences estimates and estimates adjusting for differential pre-policy trends between intervention and control counties were reported. See eFigure 1 and eFigure 2 for annual enrollment outcome trends in intervention and control counties.
4. California is the only state that has Applicable Integrated Plan (AIP) Co-D-SNPs in 2023. California's MMP demonstration ended on December 31, 2022 and beneficiaries were transitioned to D-SNPs as of January 1, 2023. Among California AIP Co-D-SNPs, 7 were identified via the CMS Plan Crosswalk as receiving MMP enrollment in 2023 and were thus classified as plans with high-level integration. See eTable 2 for list of D-SNPs identified as receiving MMP enrollment.

© 2026 Ma Y et al. *JAMA Health Forum*.

eFigure 8 Adjusted Change in Dual-eligible Enrollment Following 2023 Look-alike Plan Termination, Per Additional Percentage Point Dual-eligible Look-alike Enrollment at Baseline

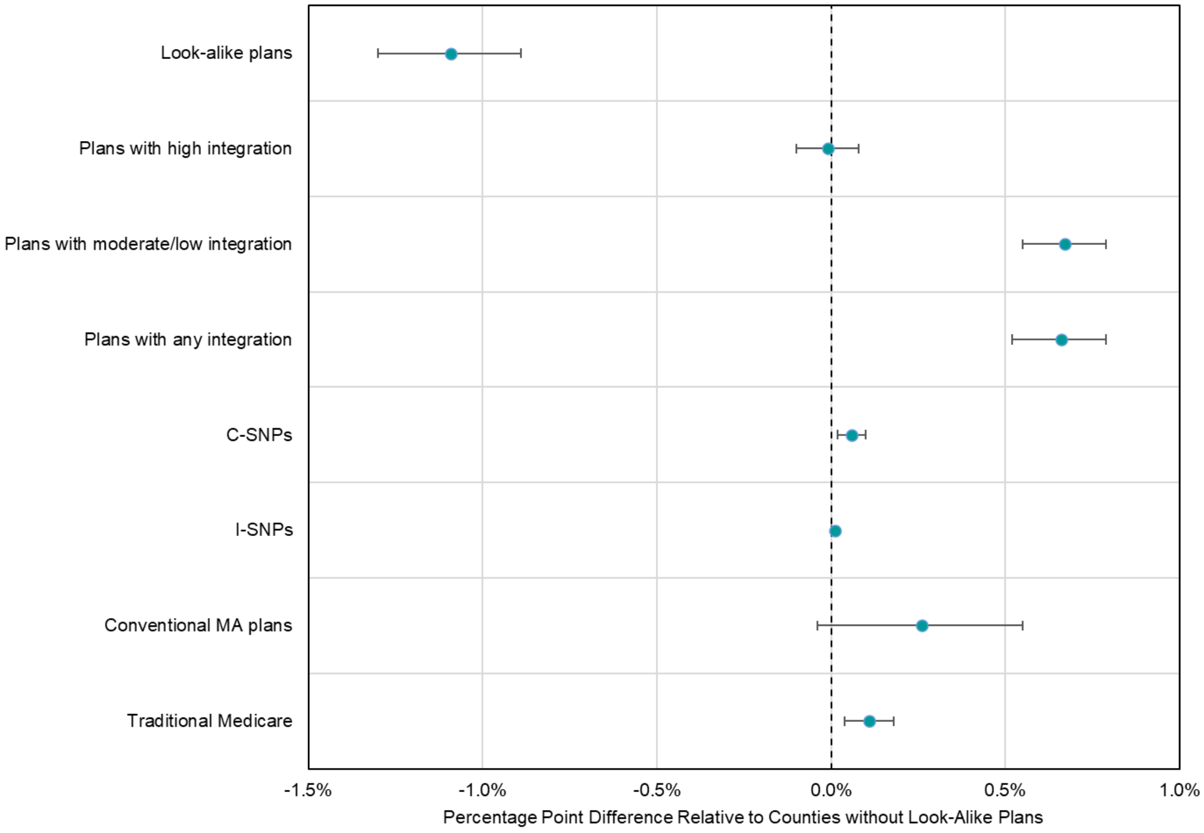

Notes:

1. Adjusted enrollment changes were estimated using a county-year-level difference-in-differences model that regressed each enrollment outcome on a post-policy year indicator, an interaction between baseline dual-eligible look-alike enrollment (measured as average proportion of dual-eligibles enrolled in look-alike plans between 2017 and 2022) and post-policy year, county fixed effects, and time-varying county-level characteristics. To address potential violations of the parallel trends assumption, the model also included a linear time trend and its interaction with the intervention county indicator to account for differential pre-policy trends between intervention and control counties. The coeffect for the interaction between baseline dual-eligible look-alike enrollment and post-policy year captured the average change in outcomes associated with the look-alike termination policy for every percentage point of dual-eligible look-alike enrollment at baseline.
2. California is the only state that has Applicable Integrated Plan (AIP) Co-D-SNPs in 2023. California's MMP demonstration ended on December 31, 2022 and beneficiaries were transitioned to D-SNPs as of January 1, 2023. Among California AIP Co-D-SNPs, 7 were identified via the CMS Plan Crosswalk as receiving MMP enrollment in 2023 and were thus classified as plans with high-level integration. See eTable 2 for list of D-SNPs identified as receiving MMP enrollment.

© 2026 Ma Y et al. *JAMA Health Forum*.

eTable 2 List of California Co-D-SNPs Identified as Receiving MMP Enrollment in January 2023

2022				2023				
Contract_ID	Plan_ID	PLAN_TYPE	No. Full-Benefit Dual-eligibles	Contract_ID	Plan_ID	PLAN_TYPE	No. Full-Benefit Dual-eligibles	Crosswalk Plan Status
H8258	1	MMP	17920	H1224	1	D-SNP	17614	Renewal Plan
H7890	1	MMP	10149	H4045	1	D-SNP	10540	Renewal Plan
H5172	2	MMP	6857	H4733	1	D-SNP	6551	Renewal Plan
H8677	1	MMP	5979	H5810	16	D-SNP	8379	Consolidated Renewal Plan
H8677	2	MMP	1337					
H7885	1	MMP	8796	H6019	1	D-SNP	8686	Renewal Plan
H5355	1	MMP	31313	H8894	1	D-SNP	31818	Renewal Plan

Notes:

1. California's MMP demonstration ended on December 31, 2022 and beneficiaries were transitioned to D-SNPs as of January 1, 2023. Among California AIP Co-D-SNPs, 7 were identified via the CMS Plan Crosswalk as receiving MMP enrollment in 2023 and were thus classified as plans with high-level integration.

© 2026 Ma Y et al. *JAMA Health Forum*.
