# Supplementary material for: Federal Look-Alike Plan Termination Policy and Dual-Eligible Enrollment in Integrated Care Programs
Source: JAMA Health Forum. 2026 Jan 16;7(1):e256294. doi: 10.1001/jamahealthforum.2025.6294 (PMC12811809; doi:10.1001/jamahealthforum.2025.6294)
Supplement: Supplement 2. — Data Sharing Statement [file jamahealthforum-e256294-s002.pdf]

Data Sharing Statement

Ma. Federal Look-Alike Plan Termination Policy and Dual-Eligible Enrollment in Integrated Care Programs. *JAMA Health Forum*. Published January 16, 2026.
doi:10.1001/jamahealthforum.2025.6294

Data
Data available: No
